# Combining plasma p‐tau_231_ and glial fibrillary acidic protein produces higher discriminative accuracy for amyloid positivity than other blood‐based biomarker combinations

**DOI:** 10.1002/alz.70796

**Published:** 2025-10-09

**Authors:** Corey J. Bolton, Omair A. Khan, Dandan Liu, Kimberly R. Pechman, Katherine A. Gifford, Timothy J. Hohman, Kaj Blennow, Henrik Zetterberg, Angela L. Jefferson

**Affiliations:** ^1^ Vanderbilt Memory and Alzheimer's Center Vanderbilt University School of Medicine Nashville Tennessee USA; ^2^ Department of Medicine Vanderbilt University Medical Center Nashville Tennessee USA; ^3^ Vanderbilt Brain Institute Vanderbilt University Nashville Tennessee USA; ^4^ Department of Biostatistics Vanderbilt University Medical Center Nashville Tennessee USA; ^5^ Department of Anatomy and Neurobiology Boston University Chobanian & Avedisian School of Medicine Boston Massachusetts USA; ^6^ Department of Neurology Vanderbilt University Medical Center Nashville Tennessee USA; ^7^ Department of Psychiatry and Neurochemistry Institute of Neuroscience and Physiology The Sahlgrenska Academy at University of Gothenburg Gothenburg Sweden; ^8^ Clinical Neurochemistry Lab Sahlgrenska University Hospital Gothenburg Sweden; ^9^ Department of Molecular Neuroscience UCL Institute of Neurology Queen Square London UK; ^10^ UK Dementia Research Institute at UCL Cruciform Building London UK; ^11^ Hong Kong Center for Neurodegenerative Diseases Hong Kong Science Park Hong Kong China; ^12^ Wisconsin Alzheimer's Disease Research Center University of Wisconsin School of Medicine and Public Health University of Wisconsin‐Madison Madison Wisconsin USA

**Keywords:** Alzheimer's disease, amyloid status, biomarkers, blood‐based biomarkers, GFAP, p‐tau_231_

## Abstract

**INTRODUCTION:**

Plasma phosphorylated tau_231_ (p‐tau_231_) has shown great promise for early identification of amyloid pathology. We tested various plasma biomarker combinations with p‐tau231 in relation to cerebrospinal fluid (CSF) amyloid positivity.

**METHODS:**

One hundred and fifty‐five dementia‐free older adults were included. Plasma p‐tau231, glial fibrillary acidic protein (GFAP), β‐amyloid42/40, and neurofilament light chain (NfL) were related to amyloid positivity via logistic regression. Subsequent models assessing various combinations of biomarkers were compared to a base model containing only p‐tau_231_.

**RESULTS:**

In single predictor models, p‐tau231 (AUC = 0.87, *p* = 0.005) and GFAP (AUC = 0.87, *p* = 0.01) were associated with amyloid positivity, and p‐tau231 remained significant in all multi‐predictor models (*p*‐values < 0.02). In comparison to the base model with p‐tau231 only, models adding GFAP improved the prediction of amyloid positivity (*p* < 0.03).

**DISCUSSION:**

Plasma p‐tau231 and GFAP were associated with amyloid positivity. Models including both p‐tau231 and GFAP performed best, while including β‐amyloid42/40 and NfL did not produce a better fitting model.

**Highlights:**

- Plasma p‐tau231 and glial fibrillary acidic protein (GFAP) are accurate predictors of amyloid positivity.
- Combining plasma p‐tau231 and GFAP improves accuracy.
- Adding other biomarkers beyond p‐tau231 and GFAP does not improve models.

## 1 INTRODUCTION

Dementia prevalence worldwide is approaching 50 million, and Alzheimer's disease (AD) dementia prevalence is expected to more than double in the United States by 2050.^1^ Novel anti‐amyloid treatments for AD are more likely to be effective in individuals without frank dementia, thus necessitating early detection in individuals who are cognitively unimpaired (CU) or only mildly symptomatic. Treatment and determination of prognosis for cognitive decline requires identification of the underlying etiology,^2^ yet determining which pathology or combination of pathologies is driving cognitive decline is challenging. Current methods of diagnosing AD in vivo, including cerebrospinal fluid (CSF) or positron emission tomography (PET) biomarkers, require specialized expertise/equipment and are not widely accessible. As such, there is a great need for an early, accurate, and accessible biomarker to screen individuals broadly for AD pathology prior to the onset of dementia.

Plasma phosphorylated tau (p‐tau) has shown great promise as a sensitive and specific biomarker for AD and is easier to obtain than CSF or PET biomarkers. Plasma p‐tau_231_, in particular, has shown a high degree of accuracy in differentiating patients with AD from controls or patients with non‐AD neurodegenerative disorders, and it is highly correlated with amyloid and tau binding on PET imaging.^3^ Plasma p‐tau_231_ reaches abnormal levels in response to a lower β‐amyloid (Aβ) burden than other blood biomarkers, including p‐tau_217_, p‐tau_181_, glial fibrillary acidic protein (GFAP), neurofilament light chain (NfL), and Aβ_42/40_ ratio.^3^, ^4^, ^5^ Additionally, plasma p‐tau_231_ is most strongly associated with amyloid PET in the earliest stages of amyloid accumulation and predicts longitudinal increases in amyloid PET uptake in individuals who are amyloid negative at baseline.^5^ Accordingly, plasma p‐tau_231_ may serve as a key indicator of the earliest stages of amyloid accumulation, prior to symptom onset, and thus play an essential role in broad screening efforts for AD pathology in asymptomatic individuals. Despite these strengths, plasma p‐tau_231_ has limitations as well. There do not seem to be longitudinal increases in plasma p‐tau_231_ over time associated with increasing Aβ burden, thus limiting its use in tracking disease progression.^4^ Further, we recently found that the sensitivity of plasma p‐tau_231_ for detecting CSF amyloid positivity was significantly reduced in individuals at later disease stages (i.e., with cognitive impairment), while sensitivity was significantly increased in individuals in the earliest disease stages (i.e., without any cognitive complaints).^6^ Given the highly specialized role that plasma p‐tau_231_ is likely to play in detecting the initial stages of amyloid accumulation, and the limitations of this biomarker in individuals with more advanced pathology, the clinical utility of this biomarker may be increased by combining it with other accessible blood biomarkers that measure unique aspects of AD pathology.

This study aims to investigate the relative value of combining plasma p‐tau_231_ with other blood biomarkers in predicting CSF amyloid positivity in dementia‐free older adults. First, we examine each plasma biomarker (p‐tau_231_, GFAP, NfL, and Aβ_42/40_) individually as a predictor of our clinically established marker, CSF amyloid positivity. Next, we examine plasma p‐tau_231_ in combination with other plasma biomarkers and perform head‐to‐head comparisons of models to determine if diagnostic accuracy is improved by the inclusion of additional unique biomarkers. Based on past work showing that plasma p‐tau_181_ and GFAP provide nonoverlapping information when combined to predict AD neuropathologic change,^7^ and a combination of plasma p‐tau_217_ and Aβ_42/40_ best predict amyloid PET positivity,^8^ we hypothesized that p‐tau_231_ combined with GFAP and Aβ_42/40_ may be most accurate at predicting CSF amyloid positivity. Finally, we repeat analyses in groups stratified by cognitive status to determine which biomarker combinations best predict amyloid positivity at each disease stage. This study provides essential information for maximizing accuracy in detecting preclinical AD with blood biomarkers, thereby increasing the feasibility of widespread screening and treatment of AD prior to the onset of clinical dementia.

## 2 METHODS

### 2.1 Study cohort

Participants were drawn from the Vanderbilt Memory and Aging Project,^9^ a longitudinal observational cohort study composed of adults aged over 60 years who are free of dementia upon study entry. Participants were required to have English language proficiency, a reliable study partner, and adequate auditory and visual acuity. At study enrollment, participants completed a clinical interview, neuropsychological assessment, and medical history review to determine study eligibility, and their cognitive status was determined by an expert consensus panel. Cognitive status was defined as CU, early mild cognitive impairment (eMCI),^10^ or mild cognitive impairment (MCI).^11^ Exclusion criteria included major psychiatric illness, metal screening contraindications for magnetic resonance imaging, a history of other neurological illness, heart failure, terminal illness, or significant head injury. Participants completed a comprehensive evaluation upon study enrollment, including (but not limited to) fasting blood draw and optional lumbar puncture. Participants were excluded from these analyses for missing plasma/CSF biomarker data and covariate data.

The Institutional Review Board of Vanderbilt University Medical Center approved the protocol. Prior to data collection, written informed consent was obtained from each participant. Protocols and data are available for sharing and may be obtained by contacting the corresponding author.

**RESEARCH IN CONTEXT**

1. **Systematic review**: The authors reviewed the literature using traditional (e.g., PubMed) sources and meeting abstracts and presentations. There have been several recent publications examining the ability of plasma phosphorylated tau 231 (p‐tau_231_) to predict amyloid positivity as a standalone biomarker. These relevant citations are appropriately cited.
2. **Interpretation**: Our findings demonstrated that the predictive accuracy of plasma p‐tau_231_ for cerebrospinal fluid amyloid positivity is improved when simultaneously considering plasma glial fibrillary acidic protein (GFAP) levels. These findings demonstrate the potential utility of including GFAP in a biomarker panel with p‐tau_231_.
3. **Future directions**: This manuscript provides key insights into the performance of readily available blood biomarkers in individuals with preclinical or prodromal Alzheimer's disease. Future studies should seek to investigate the utility of biomarker combinations in individuals with more advanced disease (i.e., dementia) to best understand which biomarkers are most useful at which disease stage.

### 2.2 Fluid collection and biochemical analysis

All participants underwent a morning fasting venous blood draw upon study entry. Plasma was isolated from ethylenediaminetetraacetic acid (EDTA) whole blood via centrifugation (2000 g at 4°C for 15 min). The lumbar puncture was performed using a polypropylene syringe with a Sprotte 25‐gauge spinal needle in an intervertebral lumbar space. Both plasma and CSF were immediately aliquoted into 0.5 mL polypropylene tubes and stored at 80°C. Biospecimens were shipped on dry ice to the Clinical Neurochemistry Laboratory at Sahlgrenska University Hospital, Mölndal, Sweden. Samples were batch analyzed by certified laboratory technicians who were blinded to clinical information. Plasma GFAP, NfL, Aβ_42_, and Aβ_40_ concentrations were measured using commercially available assays on the single‐molecule array (Simoa) HD‐X (Quanterix) analyzer (Quanterix, Billerica, MA). Plasma p‐tau_231_ concentration was measured using an in house Simoa assay as previously described.^3^ CSF samples were examined using commercially accessible enzyme‐linked immunosorbent assays (Fujirebio, Ghent, Belgium) to measure CSF concentrations of Aβ_42_ (Lumipulse G β‐Amyloid_1‐42_) and Aβ_40_ (Lumipulse G β‐Amyloid_1‐40_). For each biomarker, intraassay coefficients of variation were < 10%. CSF amyloid positivity was defined as Aβ_42/40_ ratio < 0.072.^12^

### 2.3 Covariates

Covariates, including age, sex, race/ethnicity, *apolipoprotein E (APOE*)‐ε4 carrier status, and cognitive status (as determined by Clinical Dementia Rating [CDR]^13^ global score), were collected at study entry and were determined a priori based on their potential to confound analytical models (as previously described^14^). *APOE*‐ε4 carrier status was defined as positive (ε2/ε4, ε3/ε4, ε4/ε4) or negative (ε2/ε2, ε2/ε3, ε3/ε3).

### 2.4 Analytical plan

To test the hypothesis that more abnormal blood‐biomarker levels would be associated with an increased likelihood of CSF amyloid positivity, logistic regression models related each blood biomarker (one per model) to amyloid positivity, adjusting for covariates. Subsequent models included plasma p‐tau_231_ as the predictor in a base model (adjusted for covariates) with other blood biomarkers being added first individually, then in various combinations, to the base model. Models were compared using the likelihood ratio test (LRTest) and Akaike Information Criterion (AIC) values. We then repeated analyses in groups stratified by cognitive status (CU and MCI) to determine if different biomarker combinations performed better at different disease stages.

## 3 RESULTS

### 3.1 Participant characteristics

Participants included 155 adults aged 60 to 90 years old at study entry (72 ± 6 years, 33% female, 93% non‐Hispanic White, 34% *APOE*‐ε4 carriers, 47% CSF amyloid positive). Compared to amyloid negative participants, amyloid positive participants were, on average, more likely to be female (*p* = 0.006), *APOE*‐ε4 carriers (*p* < 0.001), and cognitively impaired (*p* = 0.007). Plasma markers of p‐tau_231_ (*p* < 0.001), GFAP (*p* < 0.001), and Aβ_42/40_ ratio (*p* < 0.001) were also significantly different across groups based on amyloid positivity. See Table 1 for participant characteristics for the combined sample as well as stratified by amyloid status.

**TABLE 1:** Participant characteristics stratified by amyloid status

Parameter	Combined (*n* = 155)	Aβ (‐) (*n* = 82)	Aβ (+) (*n* = 73)	*p*‐Value^a^
Age, years	72 ± 6.3	71 ± 6.0	74 ± 6.6	0.06
Sex, % male	67	77	56	**0.006**
Education, years	16 ± 2.8	16 ± 2.9	16 ± 2.6	0.16
Race, % non‐Hispanic White	93	91	95	0.46
*APOE*‐ε4, % carrier	34	11	59	**<0.001**
MoCA, total score	25.3 ± 3.3	26.4 ± 2.6	24.9 ± .4	**0.003**
CDR global score, % normal	58	70	45	**0.007**
Plasma p‐tau_231_, pg/mL	6.7 ± 4.4	5.5 ± 3.6	8.1 ± 4.8	**<0.001**
Plasma GFAP, pg/mL	196 ± 112	154 ± 74	243 ± 129	**<0.001**
Plasma NfL, pg/mL	30 ± 21	27 ± 12	34 ± 27	0.11
Plasma Aβ_42_, pg/mL	4.8 ± 2.6	4.9 ± 2.4	4.8 ± 2.8	0.39
Plasma Aβ_40_, pg/mL	70 ± 36	68 ± 38	73 ± 34	0.73
Plasma Aβ_42/40_, ratio	0.10 ± 0.09	0.11 ± 0.09	0.09 ± 0.08	**<0.001**
CSF Aβ_42/40_, ratio	0.07 ± 0.02	0.09 ± 0.01	0.05 ± 0.0.01	**<0.001**

*Note*: Values are denoted as mean ± standard deviation or frequency. Bold font indicates *p*‐value < 0.05.

Abbreviations: Aβ, amyloid beta; *APOE*, apolipoprotein E; CDR, Clinical Dementia Rating scale; CSF, cerebrospinal fluid; GFAP, glial fibrillary acidic protein; MoCA, Montreal Cognitive Assessment; NfL, neurofilament light chain; p‐tau, phosphorylated tau.

^a^ Wilcoxon test was used for continuous variables, and Pearson's Chi‐square test was used for categorical variables.

### 3.2 Single predictor models

In single predictor models, higher plasma p‐tau_231_ (area under the receiver operating characteristic curve (AUC) = 0.87, *p* = 0.005) and GFAP (AUC = 0.87, *p* = 0.01) were associated with an increased likelihood of CSF amyloid positivity. Plasma NfL (*p* = 0.97) and Aβ_42/40_ ratio (*p* = 0.35) were not significant predictors of amyloid positivity. See Table 2 for results.

**TABLE 2:** Blood‐based biomarker associations with CSF amyloid positivity.

	AUC	AIC	*p*‐Value
**Single predictor models**			
p‐Tau_231_	0.87	148.97	**0.005**
GFAP	0.87	150.44	**0.01**
NfL	0.85	158.14	0.97
Aβ_42/40_ ratio	0.86	157.26	0.35

	p‐Tau_231_ *p*‐value	AUC	AIC	LRTest *p*‐value^a^
**Multi‐predictor models in comparison to the p‐tau_231_ base model**				
p‐Tau_231_ + GFAP	**0.007**	0.89	143.83	**0.008**
p‐Tau_231_ + NfL	**0.005**	0.87	150.96	0.90
p‐Tau_231_ + Aβ_42/40_ ratio	**0.006**	0.88	150.16	0.37
p‐Tau_231_ + GFAP + NfL	**0.008**	0.88	145.50	**0.02**
p‐Tau_231_ + GFAP + Aβ_42/40_ ratio	**0.02**	0.89	145.81	**0.03**
p‐Tau_231_ + NfL + Aβ_42/40_ ratio	**0.006**	0.88	152.13	0.65
p‐Tau_231_ + GFAP + NfL + Aβ_42/40_ ratio	**0.01**	0.89	147.40	0.06

*Note*: Models were adjusted for age, sex, race/ethnicity, *APOE*‐ε4 status, and cognitive status. Base model included p‐tau_231_ and all covariates, subsequent models (shown here) included base model plus additional blood‐biomarkers. Bold font indicates *p*‐value < 0.05.

Abbreviations: Aβ, amyloid beta; AIC, Akaike information criterion; AUC, area under the receiver operating characteristic curve; *APOE*, apolipoprotein E; CSF, cerebrospinal fluid; NfL, neurofilament light; GFAP, glial fibrillary acidic protein; NfL, neurofilament light chain; LRTest, likelihood ratio test; p‐tau, phosphorylated tau.

^a^ LRTest is comparing subsequent models to the base model.

### 3.3 Multi‐predictor models including p‐tau_231_

**FIGURE 1:**
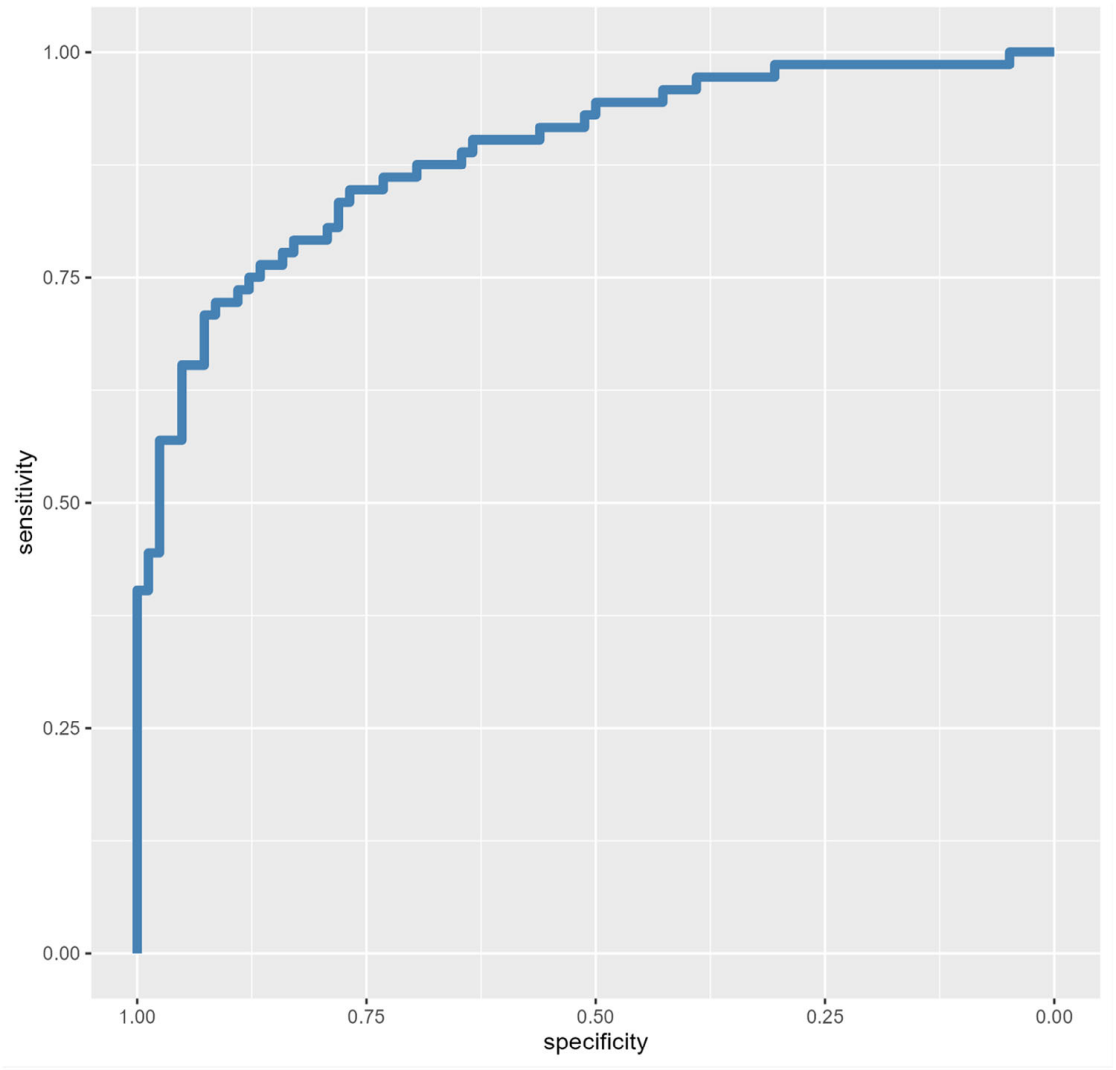
ROC curve for plasma P‐tau231 + GFAP predicting CSF amyloid positivity. Area under the ROC curve = 0.89. CSF, cerebrospinal fluid; GFAP, glial fibrillary acidic protein; p‐tau, phosphorylated tau; ROC, receiver operating characteristic.

In models including plasma p‐tau_231_ and various biomarker combinations, p‐tau_231_ remained significant in each model (*p* < 0.02). In comparison to the base model with p‐tau_231_ only, models adding GFAP (AIC = 143.8, LRTest *p* = 0.008), GFAP and NfL (AIC = 145.5, LRTest *p* = 0.02), and GFAP and Aβ_42/40_ (AIC = 145.8, LRTest *p* = 0.03) significantly improved the prediction of amyloid positivity. The model, which included GFAP, NfL, and Aβ_42/40_ in addition to p‐tau_231,_ was just beyond the threshold for statistical significance (AIC = 147.4, LRTest *p* = 0.06), and other models, which did not include GFAP, were not significantly different than the base model including just p‐tau_231_ (*p* > 0.36). The model including plasma p‐tau_231_ and GFAP was the best fitting model (AUC = 0.89), as indicated by the lowest AIC value (143.8). See Table 2 for results and Figure 1 for illustrations.

### 3.4 Stratified models by cognitive status

In CU individuals, only plasma p‐tau_231_ was a significant predictor of amyloid positivity (AUC = 0.87, *p* = 0.01), while other biomarkers were not significant (*p*‐values > 0.17). In multi‐predictor models, only the combination of p‐tau_231_, GFAP, and NfL improved upon the p‐tau_231_ single predictor model (AUC = 0.89; LRTest *p* = 0.04).

In participants with MCI, only GFAP was a significant predictor of amyloid positivity (AUC = 0.94, *p* = 0.008) while other biomarkers were not significant (*p*‐values > 0.19). In multi‐predictor models, a combination of p‐tau231 and GFAP significantly improved upon the single predictor model (AUC = 0.98, LRTest *p* = 0.003) and was the best fitting model (AIC = 48.14). See Table S1 for the results of stratified models.

## 4 DISCUSSION

In this sample of community‐dwelling older adults free of clinical dementia, we found that plasma p‐tau_231_ and plasma GFAP were strongly associated with CSF amyloid positivity. While p‐tau_231_ was the strongest single predictor of amyloid positivity among all blood‐based biomarkers examined, models that included both p‐tau_231_ and GFAP performed better than any single predictor model. The addition of other plasma biomarkers, including NfL and Aβ_42/40_, to this combination did not produce a better‐fitting model. Taken together, these findings highlight the utility of plasma p‐tau_231_ for screening for amyloid status and suggest that including plasma GFAP will improve discriminative accuracy for amyloid positivity.

Our findings highlight the value of simultaneously considering plasma p‐tau_231_ and GFAP and provide novel insights to guide clinical practice. Past work has found value in combining plasma p‐tau_231_ and GFAP in predictive models of AD neuropathologic change, noting that these biomarkers provided unique information in predicting both neuritic plaques and neurofibrillary tangle pathologies at autopsy.^7^ Our results extend this past research to show that combining these plasma biomarkers also improves the prediction of in vivo clinically established AD biomarkers. While both plasma p‐tau_231_^5^ and GFAP^15^ levels increase relatively early in the disease process following amyloid accumulation, our findings suggest that these biomarkers are unique in how they change in relation to the presence of amyloid. The phosphorylation of tau at threonine‐231 is among the earliest post‐translational modifications of tau associated with neurofibrillary tangle pathology in AD^16^ and has been linked with endogenous amyloidogenic pathways.^17^ GFAP is a marker of reactive astrocytosis, a process thought to be initiated by the formation of amyloid plaques.^18^ Simultaneously considering both protein levels can thus provide a more comprehensive picture of an individual's underlying amyloidosis and thereby improve the prediction of amyloid status. This finding has significant clinical implications as the eligibility for novel AD therapies is dependent on biomarker confirmation of amyloid status. Inaccurate frontline screening can lead to missed diagnosis, causing patients who could benefit from treatment to be deemed ineligible, or false positives, which could lead to unnecessary expenses and more invasive confirmatory procedures. As blood‐based biomarkers for AD and concomitant pathologies continue to move toward widespread clinical implementation, these results suggest that a simple combination panel of plasma p‐tau_231_ and GFAP has a high degree of concordance with a clinically established fluid biomarker, CSF Aβ_42/40_, while being more widely accessible.

In single predictor models, we found that both plasma p‐tau_231_ and GFAP were accurate predictors of CSF amyloid positivity. This finding replicates past work in European cohorts^5^, ^15^ and provides further evidence that these biomarkers are potentially valuable tools for diagnostic clarification and identification of candidates for novel anti‐amyloid therapies. Utilizing blood‐based biomarkers, such as p‐tau_231_ and GFAP, could improve accessibility of these novel treatments, as the requirement for biomarker positivity prior to initiation of treatment could exclude many patients for whom lumbar puncture or PET procedures are not accessible or available.

Plasma biomarkers of Aβ_42/40_ and NfL were not significantly associated with amyloid positivity in our analyses, likely for numerous reasons. We utilized immunoassays to quantify Aβ_42/40_. While this automated approach increases scalability, it has been shown to be less precise than other approaches, such as mass spectrometry, for quantifying Aβ_42/40_.^19^ While lacking in positive predictive value, Simoa‐based Aβ_42/40_ assays have been shown to have sufficient negative predictive value for amyloid positivity in dementia‐free older adults.^20^ Thus, the utility of this particular plasma biomarker may be highest in ruling out AD from other causes of cognitive impairment rather than definitively identifying patients with amyloidosis. NfL is a biomarker of axonal neurodegeneration, which is often a downstream event in AD but not directly associated with amyloidosis. NfL is highly accurate in distinguishing between individuals of differing cognitive status (e.g., cognitively normal vs. dementia) but does not distinguish between amyloid‐positive and amyloid‐negative participants who are asymptomatic.^21^ While not particularly helpful in detecting amyloid positivity in patients free of clinical dementia, NfL still has much value in distinguishing neurodegenerative versus non‐neurodegenerative pathologies, as well as tracking disease progression and identifying involvement of the central nervous system in other medical conditions.^22^

Although screening for amyloid positivity in asymptomatic individuals is not currently recommended in clinical practice, there remains great value in detecting amyloidosis in asymptomatic individuals for clinical trial enrollment. We found that plasma p‐tau_231_ alone was an accurate predictor of amyloid status in individuals without cognitive impairment, and adding additional biomarkers (GFAP and NfL) only marginally improved predictive accuracy. This finding suggests that plasma p‐tau_231_ could be effectively utilized as a standalone frontline screening for clinical trial enrollment. On the contrary, in participants with MCI, we found that using p‐tau_231_ alone was not a significant predictor of amyloid status, and it was necessary to add GFAP to the model to achieve the highest predictive accuracy. These findings suggest that consideration of cognitive status is essential for maximizing efficiency in blood biomarker screening for clinical trials and further highlight the need for a biomarker panel in clinical practice.

This study has numerous strengths, including the cohort used, which was composed of older adults free of dementia at study entry but enriched for amyloid positivity, thereby increasing clinical utility with a focus on individuals who may receive the most benefit from treatment. The comprehensive characterization of this cohort allows for consideration of many potential confounding variables. Biomarker quantification for both CSF and plasma was performed in batch at research‐grade laboratories with technicians who were blinded to clinical data. A notable limitation of this study is the lack of racial/ethnic diversity in the cohort, which limits the generalizability of findings. Additionally, our study does not include plasma p‐tau_217,_ which may be more predictive of amyloid pathology, especially in symptomatic individuals, than p‐tau_231_. Future work should investigate biomarker combinations with various isoforms of p‐tau.

In summary, this study replicates past work demonstrating the utility of plasma p‐tau_231_ and GFAP in predicting amyloid status in dementia‐free older adults and extends that literature by demonstrating that simultaneously considering these two biomarkers improves prediction. Other plasma biomarkers, including Aβ_42/40_ and NfL, did not add additional predictive accuracy above and beyond p‐tau_231_ single predictor models. These findings suggest that as plasma biomarkers of AD and concomitant pathologies continue to move toward clinical practice, a combination of p‐tau and GFAP biomarkers may be the best approach to identifying amyloid‐positive patients who would be potential candidates for treatment.

## CONFLICT OF INTEREST STATEMENT

T.J.H. serves on the Scientific Advisory Board for Vivid Genomics and serves as Deputy Editor for Alzheimer's and Dementia: TRCI and Senior Associate Editor for Alzheimer's and Dementia. A.L.J. has served as an advisor for Lantheus–Diagnostic and Therapeutic Innovations. K.B. has served as a consultant and on advisory boards for AbbVie, AC Immune, ALZPath, AriBio, Beckman‐Coulter, BioArctic, Biogen, Eisai, Lilly, and Moleac Pte. Ltd, Neurimmune, Novartis, Ono Pharma, Prothena, Quanterix, Roche Diagnostics, Sanofi and Siemens Healthineers; has served at data monitoring committees for Julius Clinical and Novartis; has given lectures, produced educational materials and participated in educational programs for AC Immune, Biogen, Celdara Medical, Eisai and Roche Diagnostics; and is a co‐founder of Brain Biomarker Solutions in Gothenburg AB(BBS), which is a part of the GU Ventures Incubator Program, outside the work presented in this paper. H.Z. has served at scientific advisory boards and/or as a consultant for Abbvie, Acumen, Alector, Alzinova, ALZpath, Amylyx, Annexon, Apellis, Artery Therapeutics, AZTherapies, Cognito Therapeutics, CogRx, Denali, Eisai, Enigma, LabCorp, Merry Life, Nervgen, Novo Nordisk, Optoceutics, Passage Bio, Pinteon Therapeutics, Prothena, Quanterix, Red Abbey Labs, reMYND, Roche, Samumed, Siemens Healthineers, Triplet Therapeutics, and Wave, has given lectures sponsored by Alzecure, BioArctic, Biogen, Cellectricon, Fujirebio, Lilly, Novo Nordisk, Roche, and WebMD, and is a co‐founder of Brain Biomarker Solutions in Gothenburg AB (BBS), which is a part of the GU Ventures Incubator Program (outside submitted work). C.J.B., O.A.K., D.L., K.R.P., and K.A.G. have no conflicts of interest to disclose. Author disclosures are available in the Supporting Information.

## CONSENT STATEMENT

The Vanderbilt University Medical Center Institutional Review Board granted ethical approval. All participants provided informed written consent to participate in the Vanderbilt Memory and Aging Project prior to study enrollment.

## Supporting information



Supporting information

Supporting information
