# Supplementary material for: Combining plasma p‐tau231 and glial fibrillary acidic protein produces higher discriminative accuracy for amyloid positivity than other blood‐based biomarker combinations
Source: Alzheimers Dement. 2025 Oct 9;21(10):e70796. doi: 10.1002/alz.70796 (PMC12509040; doi:10.1002/alz.70796)
Supplement: Supplementary file 1 — Supporting information [file ALZ-21-e70796-s002.docx]

| **Supplemental Table 1. Blood-Based Biomarker Associations with CSF Amyloid Positivity by Cognitive Status** | | | | | | | | |
| --- | --- | --- | --- | --- | --- | --- | --- | --- |
| *Single Predictor Models* |  |  |  | |  |  |  |  |
|  | **Cognitively Unimpaired** | | |  | **MCI** | | |  |
|  | AUC | AIC | p-value |  | AUC | AIC | p-value |  |
| P-tau_231_ | 0.87 | 82.69 | **0.01** |  | 0.90 | 55.00 | 0.19 |  |
| GFAP | 0.82 | 90.33 | 0.74 |  | 0.94 | 46.14 | **0.008** |  |
| NfL | 0.84 | 88.47 | 0.17 |  | 0.88 | 57.06 | 0.77 |  |
| Ab_42/40_ ratio | 0.83 | 88.38 | 0.17 |  | 0.88 | 57.14 | 0.92 |  |
|  |  |  |  |  |  |  |  |  |
| *Multi-Predictor Models in Comparison to P-tau_231_ Base Model* | | | | | | | | |
|  | **Cognitively Unimpaired** | | | | **MCI** | | | |
|  | p-tau_231_ p-value | AUC | AIC | LRTest p-value* | p-tau_231_ p-value | AUC | AIC | LRTest p-value* |
| P-tau_231_ + GFAP | **0.008** | 0.87 | 83.64 | 0.31 | 0.98 | 0.94 | 48.14 | **0.003** |
| P-tau_231_ + NfL | **0.007** | 0.88 | 81.75 | 0.09 | 0.18 | 0.91 | 56.76 | 0.63 |
| P-tau_231_ + Ab_42/40_ | **0.02** | 0.87 | 84.64 | 0.82 | 0.16 | 0.90 | 56.30 | 0.40 |
| P-tau_231_ + GFAP + NfL | **0.004** | 0.89 | 80.47 | **0.04** | 0.68 | 0.94 | 49.27 | **0.008** |
| P-tau_231_ + GFAP + Ab_42/40_ | **0.02** | 0.87 | 85.63 | 0.59 | 0.62 | 0.94 | 49.60 | **0.01** |
| P-tau_231_ + NfL + Ab_42/40_ | **0.01** | 0.89 | 83.12 | 0.17 | 0.16 | 0.90 | 58.28 | 0.70 |
| P-tau_231_ + GFAP + NfL + Ab_42/40_ | **0.006** | 0.90 | 81.94 | 0.08 | 0.52 | 0.93 | 51.01 | **0.02** |
| **Note.** Models were adjusted for age, sex, race/ethnicity, *APOE*-ε4 status, and cognitive status. Base model included p-tau_231_ and all covariates, subsequent models (shown here) included base model plus additional blood-biomarkers. Bold font indicates p-value<0.05. *LRTest is comparing subsequent models to the base model. Aβ, amyloid beta; AIC, Akaike information criterion; AUC, area under the receiver operating characteristic curve; *APOE*, apolipoprotein E; CSF, cerebrospinal fluid; NfL, neurofilament light; GFAP, glial fibrillary acidic protein; LRTest, likelihood ratio test; MCI, mild cognitive impairment; p-tau, phosphorylated tau. | | | | | | | | |
