# Supplementary material for: Combining plasma p‐tau231 and glial fibrillary acidic protein produces higher discriminative accuracy for amyloid positivity than other blood‐based biomarker combinations
Source: Alzheimers Dement. 2025 Oct 9;21(10):e70796. doi: 10.1002/alz.70796 (PMC12509040; doi:10.1002/alz.70796)
Supplement: Supplementary file 2 — Supporting information [file ALZ-21-e70796-s001.pdf]

# ICMJE DISCLOSURE FORM

| | |
|---|---|
| **Date:** | 9/9/2025 |
| **Your Name:** | Corey J. Bolton, PsyD |
| **Manuscript Title:** | Combining plasma p-tau231 and glial fibrillary acidic protein produces higher discriminative accuracy for amyloid positivity than other blood-based biomarker combinations |
| **Manuscript Number (if known):** | ADJ-D-25-01149 |

In the interest of transparency, we ask you to disclose all relationships/activities/interests listed below that are related to the content of your manuscript. "Related" means any relation with for-profit or not-for-profit third parties whose interests may be affected by the content of the manuscript. Disclosure represents a commitment to transparency and does not necessarily indicate a bias. If you are in doubt about whether to list a relationship/activity/interest, it is preferable that you do so.

The author's relationships/activities/interests should be defined broadly. For example, if your manuscript pertains to the epidemiology of hypertension, you should declare all relationships with manufacturers of antihypertensive medication, even if that medication is not mentioned in the manuscript.

In item #1 below, report all support for the work reported in this manuscript without time limit. For all other items, the time frame for disclosure is the past 36 months.

| | | **Name all entities with whom you have this relationship or indicate none (add rows as needed)** | **Specifications/Comments (e.g., if payments were made to you or to your institution)** |
|---|---|---|---|
| **Time frame: Since the initial planning of the work** | | | |
| **1** | All support for the present manuscript (e.g., funding, provision of study materials, medical writing, article processing charges, etc.) **No time limit for this item.** | ☐ None | |
| | | F32-AG076276 (CJB), K23-AG084850 (CJB) | |
| | | | |
| | | | Click the tab key to add additional rows. |
| **Time frame: past 36 months** | | | |
| **2** | Grants or contracts from any entity (if not indicated in item #1 above). | ☒ **None** | |
| | | | |
| | | | |
| | | | |
| **3** | Royalties or licenses | ☒ **None** | |
| | | | |
| | | | |
| | | | |

| | | Name all entities with whom you have this relationship or indicate none (add rows as needed) | Specifications/Comments (e.g., if payments were made to you or to your institution) |
|---|---|---|---|
| **4** | Consulting fees | ☒ **None** | |
| | | | |
| | | | |
| | | | |
| | | | |
| **5** | Payment or honoraria for lectures, presentations, speakers bureaus, manuscript writing or educational events | ☒ **None** | |
| | | | |
| | | | |
| | | | |
| **6** | Payment for expert testimony | ☒ **None** | |
| | | | |
| | | | |
| | | | |
| **7** | Support for attending meetings and/or travel | ☒ **None** | |
| | | | |
| | | | |
| | | | |
| **8** | Patents planned, issued or pending | ☒ **None** | |
| | | | |
| | | | |
| | | | |
| **9** | Participation on a Data Safety Monitoring Board or Advisory Board | ☒ **None** | |
| | | | |
| | | | |
| | | | |
| **10** | Leadership or fiduciary role in other board, society, committee or advocacy group, paid or unpaid | ☒ **None** | |
| | | | |
| | | | |
| | | | |

| | | Name all entities with whom you have this relationship or indicate none (add rows as needed) | Specifications/Comments (e.g., if payments were made to you or to your institution) |
|---|---|---|---|
| **11** | Stock or stock options | ☒ **None** | |
| | | | |
| | | | |
| | | | |
| **12** | Receipt of equipment, materials, drugs, medical writing, gifts or other services | ☒ **None** | |
| | | | |
| | | | |
| | | | |
| **13** | Other financial or non-financial interests | ☒ **None** | |
| | | | |
| | | | |
| | | | |

**Please place an "X" next to the following statement to indicate your agreement:**

☒ I certify that I have answered every question and have not altered the wording of any of the questions on this form.

# ICMJE DISCLOSURE FORM

| | |
|---|---|
| **Date:** | 6/23/2025 |
| **Your Name:** | Omair A. Khan |
| **Manuscript Title:** | Combining plasma p-tau231 and glial fibrillary acidic protein produces higher discriminative accuracy for amyloid positivity than other blood-based biomarker combinations |
| **Manuscript Number (if known):** | ADJ-D-25-01149 |

In the interest of transparency, we ask you to disclose all relationships/activities/interests listed below that are related to the content of your manuscript. "Related" means any relation with for-profit or not-for-profit third parties whose interests may be affected by the content of the manuscript. Disclosure represents a commitment to transparency and does not necessarily indicate a bias. If you are in doubt about whether to list a relationship/activity/interest, it is preferable that you do so.

The author's relationships/activities/interests should be defined broadly. For example, if your manuscript pertains to the epidemiology of hypertension, you should declare all relationships with manufacturers of antihypertensive medication, even if that medication is not mentioned in the manuscript.

In item #1 below, report all support for the work reported in this manuscript without time limit. For all other items, the time frame for disclosure is the past 36 months.

| | | **Name all entities with whom you have this relationship or indicate none (add rows as needed)** | **Specifications/Comments (e.g., if payments were made to you or to your institution)** |
|---|---|---|---|
| **Time frame: Since the initial planning of the work** | | | |
| **1** | All support for the present manuscript (e.g., funding, provision of study materials, medical writing, article processing charges, etc.) **No time limit for this item.** | ☒ None | |
| | | | |
| | | | |
| | | | Click the tab key to add additional rows. |
| **Time frame: past 36 months** | | | |
| **2** | Grants or contracts from any entity (if not indicated in item #1 above). | ☒ **None** | |
| | | | |
| | | | |
| | | | |
| **3** | Royalties or licenses | ☒ **None** | |
| | | | |
| | | | |
| | | | |

| | | Name all entities with whom you have this relationship or indicate none (add rows as needed) | Specifications/Comments (e.g., if payments were made to you or to your institution) |
|---|---|---|---|
| **4** | Consulting fees | ☒ **None** | |
| | | | |
| | | | |
| | | | |
| | | | |
| **5** | Payment or honoraria for lectures, presentations, speakers bureaus, manuscript writing or educational events | ☒ **None** | |
| | | | |
| | | | |
| | | | |
| **6** | Payment for expert testimony | ☒ **None** | |
| | | | |
| | | | |
| | | | |
| **7** | Support for attending meetings and/or travel | ☒ **None** | |
| | | | |
| | | | |
| | | | |
| **8** | Patents planned, issued or pending | ☒ **None** | |
| | | | |
| | | | |
| | | | |
| **9** | Participation on a Data Safety Monitoring Board or Advisory Board | ☒ **None** | |
| | | | |
| | | | |
| | | | |
| **10** | Leadership or fiduciary role in other board, society, committee or advocacy group, paid or unpaid | ☒ **None** | |
| | | | |
| | | | |
| | | | |

| | | Name all entities with whom you have this relationship or indicate none (add rows as needed) | Specifications/Comments (e.g., if payments were made to you or to your institution) |
|---|---|---|---|
| **11** | Stock or stock options | ☒ **None** | |
| | | | |
| | | | |
| | | | |
| **12** | Receipt of equipment, materials, drugs, medical writing, gifts or other services | ☒ **None** | |
| | | | |
| | | | |
| | | | |
| **13** | Other financial or non-financial interests | ☒ **None** | |
| | | | |
| | | | |
| | | | |

**Please place an "X" next to the following statement to indicate your agreement:**

☒ I certify that I have answered every question and have not altered the wording of any of the questions on this form.

# ICMJE DISCLOSURE FORM

| | |
|---|---|
| **Date:** | 7/3/2025 |
| **Your Name:** | Dandan Liu |
| **Manuscript Title:** | Combining plasma p-tau231 and glial fibrillary acidic protein produces higher discriminative accuracy for amyloid positivity than other blood-based biomarker combinations |
| **Manuscript Number (if known):** | ADJ-D-25-01149 |

In the interest of transparency, we ask you to disclose all relationships/activities/interests listed below that are related to the content of your manuscript. "Related" means any relation with for-profit or not-for-profit third parties whose interests may be affected by the content of the manuscript. Disclosure represents a commitment to transparency and does not necessarily indicate a bias. If you are in doubt about whether to list a relationship/activity/interest, it is preferable that you do so.

The author's relationships/activities/interests should be defined broadly. For example, if your manuscript pertains to the epidemiology of hypertension, you should declare all relationships with manufacturers of antihypertensive medication, even if that medication is not mentioned in the manuscript.

In item #1 below, report all support for the work reported in this manuscript without time limit. For all other items, the time frame for disclosure is the past 36 months.

| | | **Name all entities with whom you have this relationship or indicate none (add rows as needed)** | **Specifications/Comments (e.g., if payments were made to you or to your institution)** |
|---|---|---|---|
| **Time frame: Since the initial planning of the work** | | | |
| **1** | All support for the present manuscript (e.g., funding, provision of study materials, medical writing, article processing charges, etc.) **No time limit for this item.** | ☒ None | |
| | | | |
| | | | |
| | | | Click the tab key to add additional rows. |
| **Time frame: past 36 months** | | | |
| **2** | Grants or contracts from any entity (if not indicated in item #1 above). | ☒ **None** | |
| | | | |
| | | | |
| | | | |
| **3** | Royalties or licenses | ☒ **None** | |
| | | | |
| | | | |
| | | | |

| | | Name all entities with whom you have this relationship or indicate none (add rows as needed) | Specifications/Comments (e.g., if payments were made to you or to your institution) |
|---|---|---|---|
| **4** | Consulting fees | ☒ **None** | |
| | | | |
| | | | |
| | | | |
| | | | |
| **5** | Payment or honoraria for lectures, presentations, speakers bureaus, manuscript writing or educational events | ☒ **None** | |
| | | | |
| | | | |
| | | | |
| **6** | Payment for expert testimony | ☒ **None** | |
| | | | |
| | | | |
| | | | |
| **7** | Support for attending meetings and/or travel | ☒ **None** | |
| | | | |
| | | | |
| | | | |
| **8** | Patents planned, issued or pending | ☒ **None** | |
| | | | |
| | | | |
| | | | |
| **9** | Participation on a Data Safety Monitoring Board or Advisory Board | ☒ **None** | |
| | | | |
| | | | |
| | | | |
| **10** | Leadership or fiduciary role in other board, society, committee or advocacy group, paid or unpaid | ☒ **None** | |
| | | | |
| | | | |
| | | | |

| | | Name all entities with whom you have this relationship or indicate none (add rows as needed) | Specifications/Comments (e.g., if payments were made to you or to your institution) |
|---|---|---|---|
| **11** | Stock or stock options | ☒ **None** | |
| | | | |
| | | | |
| | | | |
| **12** | Receipt of equipment, materials, drugs, medical writing, gifts or other services | ☒ **None** | |
| | | | |
| | | | |
| | | | |
| **13** | Other financial or non-financial interests | ☒ **None** | |
| | | | |
| | | | |
| | | | |

**Please place an "X" next to the following statement to indicate your agreement:**

☒ I certify that I have answered every question and have not altered the wording of any of the questions on this form.

# ICMJE DISCLOSURE FORM

| | |
|---|---|
| **Date:** | 6/25/2025 |
| **Your Name:** | Kimberly R. Pechman |
| **Manuscript Title:** | Combining plasma p-tau231 and glial fibrillary acidic protein produces higher discriminative accuracy for amyloid positivity than other blood-based biomarker combinations |
| **Manuscript Number (if known):** | ADJ-D-25-01149 |

In the interest of transparency, we ask you to disclose all relationships/activities/interests listed below that are related to the content of your manuscript. "Related" means any relation with for-profit or not-for-profit third parties whose interests may be affected by the content of the manuscript. Disclosure represents a commitment to transparency and does not necessarily indicate a bias. If you are in doubt about whether to list a relationship/activity/interest, it is preferable that you do so.

The author's relationships/activities/interests should be defined broadly. For example, if your manuscript pertains to the epidemiology of hypertension, you should declare all relationships with manufacturers of antihypertensive medication, even if that medication is not mentioned in the manuscript.

In item #1 below, report all support for the work reported in this manuscript without time limit. For all other items, the time frame for disclosure is the past 36 months.

| | | Name all entities with whom you have this relationship or indicate none (add rows as needed) | Specifications/Comments (e.g., if payments were made to you or to your institution) |
|---|---|---|---|
| Time frame: Since the initial planning of the work | | | |
| 1 | All support for the present manuscript (e.g., funding, provision of study materials, medical writing, article processing charges, etc.) **No time limit for this item.** | ☒ None | Click the tab key to add additional rows. |
| Time frame: past 36 months | | | |
| 2 | Grants or contracts from any entity (if not indicated in item #1 above). | ☒ **None** | |
| 3 | Royalties or licenses | ☒ **None** | |

| | | Name all entities with whom you have this relationship or indicate none (add rows as needed) | Specifications/Comments (e.g., if payments were made to you or to your institution) |
|---|---|---|---|
| **4** | Consulting fees | ☒ **None** | |
| | | | |
| | | | |
| | | | |
| | | | |
| **5** | Payment or honoraria for lectures, presentations, speakers bureaus, manuscript writing or educational events | ☒ **None** | |
| | | | |
| | | | |
| | | | |
| **6** | Payment for expert testimony | ☒ **None** | |
| | | | |
| | | | |
| | | | |
| **7** | Support for attending meetings and/or travel | ☒ **None** | |
| | | | |
| | | | |
| | | | |
| **8** | Patents planned, issued or pending | ☒ **None** | |
| | | | |
| | | | |
| | | | |
| **9** | Participation on a Data Safety Monitoring Board or Advisory Board | ☒ **None** | |
| | | | |
| | | | |
| | | | |
| **10** | Leadership or fiduciary role in other board, society, committee or advocacy group, paid or unpaid | ☒ **None** | |
| | | | |
| | | | |
| | | | |

| | | Name all entities with whom you have this relationship or indicate none (add rows as needed) | Specifications/Comments (e.g., if payments were made to you or to your institution) |
|---|---|---|---|
| **11** | Stock or stock options | ☒ **None** | |
| | | | |
| | | | |
| | | | |
| **12** | Receipt of equipment, materials, drugs, medical writing, gifts or other services | ☒ **None** | |
| | | | |
| | | | |
| | | | |
| **13** | Other financial or non-financial interests | ☒ **None** | |
| | | | |
| | | | |
| | | | |

**Please place an "X" next to the following statement to indicate your agreement:**

☒ I certify that I have answered every question and have not altered the wording of any of the questions on this form.

# ICMJE DISCLOSURE FORM

**Date:** 6/23/2025

**Your Name:** Katherine Gifford, PsyD

**Manuscript Title:** Combining plasma p-tau231 and glial fibrillary acidic protein produces higher discriminative accuracy for amyloid positivity than other blood-based biomarker combinations

**Manuscript Number (if known):** ADJ-D-25-01149

In the interest of transparency, we ask you to disclose all relationships/activities/interests listed below that are related to the content of your manuscript. "Related" means any relation with for-profit or not-for-profit third parties whose interests may be affected by the content of the manuscript. Disclosure represents a commitment to transparency and does not necessarily indicate a bias. If you are in doubt about whether to list a relationship/activity/interest, it is preferable that you do so.

The author's relationships/activities/interests should be defined broadly. For example, if your manuscript pertains to the epidemiology of hypertension, you should declare all relationships with manufacturers of antihypertensive medication, even if that medication is not mentioned in the manuscript.

In item #1 below, report all support for the work reported in this manuscript without time limit. For all other items, the time frame for disclosure is the past 36 months.

| | | Name all entities with whom you have this relationship or indicate none (add rows as needed) | Specifications/Comments (e.g., if payments were made to you or to your institution) |
|---|---|---|---|
| **Time frame: Since the initial planning of the work** | | | |
| 1 | All support for the present manuscript (e.g., funding, provision of study materials, medical writing, article processing charges, etc.) **No time limit for this item.** | ☐ None | |
| | | R01 AG062826] | Paid to institution |
| | | | |
| | | | Click the tab key to add additional rows. |
| **Time frame: past 36 months** | | | |
| **2** | Grants or contracts from any entity (if not indicated in item #1 above). | ☒ **None** | |
| | | | |
| | | | |
| | | | |
| **3** | Royalties or licenses | ☒ **None** | |
| | | | |
| | | | |
| | | | |

| | | Name all entities with whom you have this relationship or indicate none (add rows as needed) | Specifications/Comments (e.g., if payments were made to you or to your institution) |
|---|---|---|---|
| **4** | Consulting fees | ☒ **None** | |
| | | | |
| | | | |
| | | | |
| | | | |
| **5** | Payment or honoraria for lectures, presentations, speakers bureaus, manuscript writing or educational events | ☒ **None** | |
| | | | |
| | | | |
| | | | |
| **6** | Payment for expert testimony | ☒ **None** | |
| | | | |
| | | | |
| | | | |
| **7** | Support for attending meetings and/or travel | ☒ **None** | |
| | | | |
| | | | |
| | | | |
| **8** | Patents planned, issued or pending | ☒ **None** | |
| | | | |
| | | | |
| | | | |
| **9** | Participation on a Data Safety Monitoring Board or Advisory Board | ☒ **None** | |
| | | | |
| | | | |
| | | | |
| **10** | Leadership or fiduciary role in other board, society, committee or advocacy group, paid or unpaid | ☒ **None** | |
| | | | |
| | | | |
| | | | |

| | | Name all entities with whom you have this relationship or indicate none (add rows as needed) | Specifications/Comments (e.g., if payments were made to you or to your institution) |
|---|---|---|---|
| **11** | Stock or stock options | ☒ **None** | |
| | | | |
| | | | |
| | | | |
| **12** | Receipt of equipment, materials, drugs, medical writing, gifts or other services | ☒ **None** | |
| | | | |
| | | | |
| | | | |
| **13** | Other financial or non-financial interests | ☒ **None** | |
| | | | |
| | | | |
| | | | |

**Please place an "X" next to the following statement to indicate your agreement:**

☒ I certify that I have answered every question and have not altered the wording of any of the questions on this form.

# ICMJE DISCLOSURE FORM

**Date:** 8/4/2025

**Your Name:** Timothy Hohman

**Manuscript Title:** Combining plasma p-tau231 and glial fibrillary acidic protein produces higher discriminative accuracy for amyloid positivity than other blood-based biomarker combinations

**Manuscript Number (if known):** ADJ-D-25-01149

In the interest of transparency, we ask you to disclose all relationships/activities/interests listed below that are related to the content of your manuscript. "Related" means any relation with for-profit or not-for-profit third parties whose interests may be affected by the content of the manuscript. Disclosure represents a commitment to transparency and does not necessarily indicate a bias. If you are in doubt about whether to list a relationship/activity/interest, it is preferable that you do so.

The author's relationships/activities/interests should be defined broadly. For example, if your manuscript pertains to the epidemiology of hypertension, you should declare all relationships with manufacturers of antihypertensive medication, even if that medication is not mentioned in the manuscript.

In item #1 below, report all support for the work reported in this manuscript without time limit. For all other items, the time frame for disclosure is the past 36 months.

| | | **Name all entities with whom you have this relationship or indicate none (add rows as needed)** | **Specifications/Comments (e.g., if payments were made to you or to your institution)** |
|---|---|---|---|
| **Time frame: Since the initial planning of the work** | | | |
| 1 | All support for the present manuscript (e.g., funding, provision of study materials, medical writing, article processing charges, etc.) **No time limit for this item.** | ☐ None | |
| | | National Institute of Health | Grants |
| | | | |
| | | | Click the tab key to add additional rows. |
| **Time frame: past 36 months** | | | |
| 2 | Grants or contracts from any entity (if not indicated in item #1 above). | ☒ **None** | |
| | | | |
| | | | |
| | | | |
| 3 | Royalties or licenses | ☒ **None** | |
| | | | |
| | | | |
| | | | |

| | | Name all entities with whom you have this relationship or indicate none (add rows as needed) | Specifications/Comments (e.g., if payments were made to you or to your institution) |
|---|---|---|---|
| **4** | Consulting fees | ☐ **None** | |
| | | Circular Genomics | Consultant |
| | | | |
| | | | |
| | | | |
| **5** | Payment or honoraria for lectures, presentations, speakers bureaus, manuscript writing or educational events | ☒ **None** | |
| | | | |
| | | | |
| | | | |
| **6** | Payment for expert testimony | ☒ **None** | |
| | | | |
| | | | |
| | | | |
| **7** | Support for attending meetings and/or travel | ☐ **None** | |
| | | Alzheimer's Association | |
| | | | |
| | | | |
| **8** | Patents planned, issued or pending | ☒ **None** | |
| | | | |
| | | | |
| | | | |
| **9** | Participation on a Data Safety Monitoring Board or Advisory Board | ☐ **None** | |
| | | Vivid Genomics | Scientific Advisory Board |
| | | Fidelity Foundation | Scientific Advisory Board |
| | | | |
| **10** | Leadership or fiduciary role in other board, society, committee or advocacy group, paid or unpaid | ☒ **None** | |
| | | | |
| | | | |
| | | | |

| | | Name all entities with whom you have this relationship or indicate none (add rows as needed) | Specifications/Comments (e.g., if payments were made to you or to your institution) |
|---|---|---|---|
| **11** | Stock or stock options | ☐ **None** | |
| | | Vivid Genomics | Scientific Advisory Board |
| | | | |
| | | | |
| **12** | Receipt of equipment, materials, drugs, medical writing, gifts or other services | ☒ **None** | |
| | | | |
| | | | |
| | | | |
| **13** | Other financial or non-financial interests | ☐ **None** | |
| | | Alzheimer's Association | Deputy Editor for the Alzheimer's & Dementia: TRCI |
| | | Alzheimer's Association | Senior Associate Editor for Alzheimer's & Dementia |
| | | | |

**Please place an "X" next to the following statement to indicate your agreement:**

☒ I certify that I have answered every question and have not altered the wording of any of the questions on this form.

# ICMJE DISCLOSURE FORM

**Date:** 9/9/2025

**Your Name:** Kaj Blennow

**Manuscript Title:** Combining plasma p-tau231 and glial fibrillary acidic protein produces higher discriminative accuracy for amyloid positivity than other blood-based biomarker combinations

**Manuscript Number (if known):** ADJ-D-25-01149

In the interest of transparency, we ask you to disclose all relationships/activities/interests listed below that are related to the content of your manuscript. "Related" means any relation with for-profit or not-for-profit third parties whose interests may be affected by the content of the manuscript. Disclosure represents a commitment to transparency and does not necessarily indicate a bias. If you are in doubt about whether to list a relationship/activity/interest, it is preferable that you do so.

The author's relationships/activities/interests should be defined broadly. For example, if your manuscript pertains to the epidemiology of hypertension, you should declare all relationships with manufacturers of antihypertensive medication, even if that medication is not mentioned in the manuscript.

In item #1 below, report all support for the work reported in this manuscript without time limit. For all other items, the time frame for disclosure is the past 36 months.

| | | Name all entities with whom you have this relationship or indicate none (add rows as needed) | Specifications/Comments (e.g., if payments were made to you or to your institution) |
|---|---|---|---|
| Time frame: Since the initial planning of the work | | | |
| 1 | All support for the present manuscript (e.g., funding, provision of study materials, medical writing, article processing charges, etc.)<br>No time limit for this item. | ☐ None<br><br>KB is supported by the Swedish Research Council (#2017-00915 and #2022-00732), the Swedish Alzheimer Foundation (#AF-930351, #AF-939721, #AF-968270, and #AF-994551), Hjärnfonden, Sweden (#ALZ2022-0006, #FO2024-0048-TK-130 and FO2024-0048-HK-24), the Swedish state under the agreement between the Swedish government and the County Councils, the ALF-agreement (#ALFGBG-965240 and #ALFGBG-1006418), the European Union Joint Program for Neurodegenerative Disorders (JPND2019-466-236), the Alzheimer's Association 2021 Zenith Award (ZEN-21-848495), the Alzheimer's Association 2022-2025 Grant (SG-23-1038904 QC), La Fondation Recherche Alzheimer (FRA), Paris, France, the Kirsten and Freddy Johansen Foundation, Copenhagen, Denmark, Familjen | |

| | | Name all entities with whom you have this relationship or indicate none (add rows as needed) | Specifications/Comments (e.g., if payments were made to you or to your institution) |
|---|---|---|---|
| | | Rönströms Stiftelse, Stockholm, Sweden, and an anonymous filantropist and donor. | |
| | | KB has served as a consultant and at advisory boards for Abbvie, AC Immune, ALZPath, AriBio, Beckman-Coulter, BioArctic, Biogen, Eisai, Lilly, Moleac Pte. Ltd, Neurimmune, Novartis, Ono Pharma, Prothena, Quanterix, Roche Diagnostics, Sanofi and Siemens Healthineers; has served at data monitoring committees for Julius Clinical and Novartis; has given lectures, produced educational materials and participated in educational programs for AC Immune, Biogen, Celdara Medical, Eisai and Roche Diagnostics; and is a co-founder of Brain Biomarker Solutions in Gothenburg AB(BBS), which is a part of the GU Ventures Incubator Program, outside the work presented in this paper | |
| | | | Click the tab key to add additional rows. |
| Time frame: past 36 months | | | |
| **2** | Grants or contracts from any entity (if not indicated in item #1 above). | ☒ **None** | |
| | | | |
| | | | |
| | | | |
| **3** | Royalties or licenses | ☒ **None** | |
| | | | |
| | | | |
| | | | |
| **4** | Consulting fees | ☒ **None** | |
| | | | |
| | | | |
| | | | |
| | | | |
| **5** | Payment or honoraria for lectures, presentations, speakers bureaus, manuscript writing or educational events | ☒ **None** | |
| | | | |
| | | | |
| | | | |

| | | Name all entities with whom you have this relationship or indicate none (add rows as needed) | Specifications/Comments (e.g., if payments were made to you or to your institution) |
|---|---|---|---|
| **6** | Payment for expert testimony | ☒ **None** | |
| | | | |
| | | | |
| | | | |
| **7** | Support for attending meetings and/or travel | ☒ **None** | |
| | | | |
| | | | |
| | | | |
| **8** | Patents planned, issued or pending | ☒ **None** | |
| | | | |
| | | | |
| | | | |
| **9** | Participation on a Data Safety Monitoring Board or Advisory Board | ☒ **None** | |
| | | | |
| | | | |
| | | | |
| **10** | Leadership or fiduciary role in other board, society, committee or advocacy group, paid or unpaid | ☒ **None** | |
| | | | |
| | | | |
| | | | |
| **11** | Stock or stock options | ☒ **None** | |
| | | | |
| | | | |
| | | | |
| **12** | Receipt of equipment, materials, drugs, medical writing, gifts or other services | ☒ **None** | |
| | | | |
| | | | |
| | | | |
| **13** | Other financial or non-financial interests | ☒ **None** | |
| | | | |
| | | | |
| | | | |

| | Name all entities with whom you have this relationship or indicate none (add rows as needed) | Specifications/Comments (e.g., if payments were made to you or to your institution) |
|---|---|---|

**Please place an "X" next to the following statement to indicate your agreement:**

☒ I certify that I have answered every question and have not altered the wording of any of the questions on this form.

**ICMJE DISCLOSURE FORM**

**Date:**__________2025-06-21___________________________________________________________
**Your Name: Henrik Zetterberg**____________________________________________________
**Manuscript Title: Combining plasma p-tau231 and glial fibrillary acidic protein produces higher discriminative accuracy for amyloid positivity than other blood-based biomarker combinations**
**Manuscript number (if known): ADJ-D-25-01149**

**In the interest of transparency, we ask you to disclose all relationships/activities/interests listed below that are related to the content of your manuscript. "Related" means any relation with for-profit or not-for-profit third parties whose interests may be affected by the content of the manuscript. Disclosure represents a commitment to transparency and does not necessarily indicate a bias. If you are in doubt about whether to list a relationship/activity/interest, it is preferable that you do so.**

**The following questions apply to the author's relationships/activities/interests as they relate to the current manuscript only.**

**The author's relationships/activities/interests should be defined broadly. For example, if your manuscript pertains to the epidemiology of hypertension, you should declare all relationships with manufacturers of antihypertensive medication, even if that medication is not mentioned in the manuscript.**

**In item #1 below, report all support for the work reported in this manuscript without time limit. For all other items, the time frame for disclosure is the past 36 months.**

| | | Name all entities with whom you have this relationship or indicate none (add rows as needed) | Specifications/Comments (e.g., if payments were made to you or to your institution) |
|---|---|---|---|
| | | **Time frame: Since the initial planning of the work** | |
| 1 | All support for the present manuscript (e.g., funding, provision of study materials, medical writing, article processing charges, etc.) | ☐ **None** | |
| | | HZ is a Wallenberg Scholar and a Distinguished Professor at the Swedish Research Council supported by grants from the Swedish Research Council (#2023-00356, #2022-01018 and #2019-02397), the European Union's Horizon Europe research and innovation programme under grant agreement No 101053962, Swedish State Support for Clinical Research (#ALFGBG-71320), the Alzheimer Drug Discovery Foundation (ADDF), USA (#201809-2016862), the AD Strategic Fund and the Alzheimer's Association (#ADSF-21-831376-C, #ADSF-21-831381-C, #ADSF-21-831377-C, and #ADSF-24-1284328-C), the European Partnership on Metrology, co-financed from the European Union's Horizon Europe Research and Innovation Programme and by the | Payments made to Institution. |

| | | Name all entities with whom you have this relationship or indicate none (add rows as needed) | Specifications/Comments (e.g., if payments were made to you or to your institution) |
|---|---|---|---|
| | **No time limit for this item.** | Participating States (NEuroBioStand, #22HLT07), the Bluefield Project, Cure Alzheimer's Fund, the Olav Thon Foundation, the Erling-Persson Family Foundation, Familjen Rönströms Stiftelse, Familjen Beiglers Stiftelse, Stiftelsen för Gamla Tjänarinnor, Hjärnfonden, Sweden (#FO2022-0270), the European Union's Horizon 2020 research and innovation programme under the Marie Skłodowska-Curie grant agreement No 860197 (MIRIADE), the European Union Joint Programme – Neurodegenerative Disease Research (JPND2021-00694), the National Institute for Health and Care Research University College London Hospitals Biomedical Research Centre, the UK Dementia Research Institute at UCL (UKDRI-1003), and an anonymous donor. | |
| | | | |
| | | | Click the tab key to add additional rows. |
| **Time frame: past 36 months** | | | |
| **2** | Grants or contracts from any entity (if not indicated in item #1 above). | ☐ **None** | |
| | | HZ is a Wallenberg Scholar and a Distinguished Professor at the Swedish Research Council supported by grants from the Swedish Research Council (#2023-00356, #2022-01018 and #2019-02397), the European Union's Horizon Europe research and innovation programme under grant agreement No 101053962, Swedish State Support for Clinical Research (#ALFGBG-71320), the Alzheimer Drug Discovery Foundation (ADDF), USA (#201809-2016862), the AD Strategic Fund and the Alzheimer's Association (#ADSF-21-831376-C, #ADSF-21-831381-C, #ADSF-21-831377-C, and #ADSF-24-1284328-C), the European Partnership on Metrology, co-financed from the European Union's Horizon Europe Research and Innovation | Payments made to Institu |

| | | Name all entities with whom you have this relationship or indicate none (add rows as needed) | Specifications/Comments (e.g., if payments were made to you or to your institution) |
|---|---|---|---|
| | | Programme and by the Participating States (NEuroBioStand, #22HLT07), the Bluefield Project, Cure Alzheimer's Fund, the Olav Thon Foundation, the Erling-Persson Family Foundation, Familjen Rönströms Stiftelse, Familjen Beiglers Stiftelse, Stiftelsen för Gamla Tjänarinnor, Hjärnfonden, Sweden (#FO2022-0270), the European Union's Horizon 2020 research and innovation programme under the Marie Skłodowska-Curie grant agreement No 860197 (MIRIADE), the European Union Joint Programme – Neurodegenerative Disease Research (JPND2021-00694), the National Institute for Health and Care Research University College London Hospitals Biomedical Research Centre, the UK Dementia Research Institute at UCL (UKDRI-1003), and an anonymous donor. | |
| | | | |
| | | | |
| **3** | Royalties or licenses | ☒ **None** | |
| | | | |
| | | | |
| | | | |
| **4** | Consulting fees | ☐ **None** | |
| | | HZ has served at scientific advisory boards and/or as a consultant for Abbvie, Acumen, Alector, Alzinova, ALZpath, Amylyx, Annexon, Apellis, Artery Therapeutics, AZTherapies, Cognito Therapeutics, CogRx, Denali, Eisai, Enigma, LabCorp, Merck Sharp & | Payments made to HZ. |

| | | Name all entities with whom you have this relationship or indicate none (add rows as needed) | Specifications/Comments (e.g., if payments were made to you or to your institution) |
|---|---|---|---|
| | | Dohme, Merry Life, Nervgen, Novo Nordisk, Optoceutics, Passage Bio, Pinteon Therapeutics, Prothena, Quanterix, Red Abbey Labs, reMYND, Roche, Samumed, ScandiBio Therapeutics AB, Siemens Healthineers, Triplet Therapeutics, and Wave. | |
| | | | |
| | | | |
| | | | |
| **5** | Payment or honoraria for lectures, presentations, speakers bureaus, manuscript writing or educational events | ☐ **None** | |
| | | HZ has given lectures sponsored by Alzecure, BioArctic, Biogen, Cellectricon, Fujirebio, LabCorp, Lilly, Novo Nordisk, Oy Medix Biochemica AB, Roche, and WebMD. | Payments made to HZ. |
| | | | |
| | | | |
| **6** | Payment for expert testimony | ☒ **None** | |
| | | | |
| | | | |
| | | | |
| **7** | Support for attending meetings and/or travel | ☒ **None** | |
| | | | |
| | | | |
| | | | |

| | | Name all entities with whom you have this relationship or indicate none (add rows as needed) | Specifications/Comments (e.g., if payments were made to you or to your institution) |
|---|---|---|---|
| **8** | Patents planned, issued or pending | ☒ **None** | |
| | | | |
| | | | |
| | | | |
| **9** | Participation on a Data Safety Monitoring Board or Advisory Board | ☐ **None** | |
| | | HZ has served at scientific advisory boards and/or as a consultant for Abbvie, Acumen, Alector, Alzinova, ALZpath, Amylyx, Annexon, Apellis, Artery Therapeutics, AZTherapies, Cognito Therapeutics, CogRx, Denali, Eisai, Enigma, LabCorp, Merck Sharp & Dohme, Merry Life, Nervgen, Novo Nordisk, Optoceutics, Passage Bio, Pinteon Therapeutics, Prothena, Quanterix, Red Abbey Labs, reMYND, Roche, Samumed, ScandiBio Therapeutics AB, Siemens Healthineers, Triplet Therapeutics, and Wave. | Payments made to HZ. |
| | | | |
| | | | |
| **10** | Leadership or fiduciary role in other board, society, committee or advocacy group, paid or unpaid | ☐ **None** | |
| | | HZ is chair of the Alzheimer's Association Global Biomarker Standardization Consortium and chair of the IFCC WG-BND. | No payments made. |
| | | | |
| | | | |

| | | Name all entities with whom you have this relationship or indicate none (add rows as needed) | Specifications/Comments (e.g., if payments were made to you or to your institution) |
|---|---|---|---|
| 11 | Stock or stock options | ☐ None | |
| | | HZ is a co-founder of Brain Biomarker Solutions in Gothenburg AB (BBS), which is a part of the GU Ventures Incubator Program, and a shareholder of MicThera (outside submitted work) | Payments made to HZ. |
| | | | |
| | | | |
| 12 | Receipt of equipment, materials, drugs, medical writing, gifts or other services | ☒ None | |
| | | | |
| | | | |
| | | | |
| 13 | Other financial or non-financial interests | ☒ None | |
| | | | |
| | | | |
| | | | |

**Please place an "X" next to the following statement to indicate your agreement:**

☒ I certify that I have answered every question and have not altered the wording of any of the questions on this form.

# ICMJE DISCLOSURE FORM

**Date:** 6/20/2025

**Your Name:** Dr. Angela L. Jefferson

**Manuscript Title:** Combining plasma p-tau231 and glial fibrillary acidic protein produces higher discriminative accuracy for amyloid positivity than other blood-based biomarker combinations

**Manuscript Number (if known):** ADJ-D-25-01149

In the interest of transparency, we ask you to disclose all relationships/activities/interests listed below that are related to the content of your manuscript. "Related" means any relation with for-profit or not-for-profit third parties whose interests may be affected by the content of the manuscript. Disclosure represents a commitment to transparency and does not necessarily indicate a bias. If you are in doubt about whether to list a relationship/activity/interest, it is preferable that you do so.

The author's relationships/activities/interests should be defined broadly. For example, if your manuscript pertains to the epidemiology of hypertension, you should declare all relationships with manufacturers of antihypertensive medication, even if that medication is not mentioned in the manuscript.

In item #1 below, report all support for the work reported in this manuscript without time limit. For all other items, the time frame for disclosure is the past 36 months.

| | | **Name all entities with whom you have this relationship or indicate none (add rows as needed)** | **Specifications/Comments (e.g., if payments were made to you or to your institution)** |
|---|---|---|---|
| **Time frame: Since the initial planning of the work** | | | |
| 1 | All support for the present manuscript (e.g., funding, provision of study materials, medical writing, article processing charges, etc.)<br>No time limit for this item. | ☐ None | |
| | | NIH/NIA R01-AG034962 | Project Role: Principal Investigator<br>Title: The Vanderbilt Memory and Aging Project<br>Award period: 03/14/11-03/31/26 |
| | | NIH/NIA R01-AG056534 | Project Role: Principal Investigator<br>Title: White matter and small vessel disease in older adults<br>Award period: 09/15/17-05/31/23 |
| | | NIH/NIA K24-AG046373 | Project Role: Principal Investigator<br>Title: Risk factors and prevention targets for abnormal cognitive aging<br>Award period: 12/01/13-12/31/24 |
| | | Alzheimer's Association Investigator Initiated Research IIRG-08-88733 | Project Role: Principal Investigator<br>Title: Left ventricular function & mild cognitive impairment<br>Award period: 08/01/08-07/31/14 |
| **Time frame: past 36 months** | | | |
| **2** | Grants or contracts from any entity (if not indicated in item #1 above). | ☐ **None** | |
| | | NIH/NINDS R01-NS100980 | Project Role: Principal Investigator<br>Title: Cardiovascular predictors of cerebrovascular health in older adults<br>Award period: 09/01/17-06/30/23 |

| | | Name all entities with whom you have this relationship or indicate none (add rows as needed) | Specifications/Comments (e.g., if payments were made to you or to your institution) |
|---|---|---|---|
| | | NIH/NIA P20-AG068082 | Project Role: Principal Investigator, Center Director<br>Title: Vanderbilt Alzheimer's Disease Research Center<br>Award period: 08/15/20-07/31/25 |
| | | NIH/NIA T32-AG058524 | Project Role: Principal Investigator<br>Title: Vanderbilt Interdisciplinary Training Program in Alzheimer's Disease<br>Award period: 05/01/18-04/30/29 |
| | | NIH/NIA R01-AG058639<br>(MPI: Ely, Patel, Jefferson) | Project Role: Multi-Principal Investigator<br>Title: BRAIN-ICU-2 Study: Bringing to light the risk factors and incidence of neuropsychological dysfunction (dementia) in ICU survivors, 2nd study<br>Award period: 04/15/19-01/31/25 |
| | | NIH/NIBIB R01-EB017230<br>(PI: Landman) | Project Role: Site Principal Investigator<br>Title: Controlling quality and capturing uncertainty in advanced diffusion weighted MRI<br>Award period: 10/01/21-9/30/26 |
| | | Biohaven Pharmaceuticals | Project Role: Site Principal Investigator<br>Title: A phase II randomized double-blind placebo controlled trial to evaluate the efficacy and safety of BHV-4157 in patients with mild to moderate Alzheimer's disease<br>Award period: 12/01/18-06/30/22 |
| **3** | Royalties or licenses | ☒ **None** | |
| | | | |
| | | | |
| | | | |
| **4** | Consulting fees | ☒ **None** | |
| | | | |
| | | | |
| | | | |
| | | | |
| **5** | Payment or honoraria for lectures, presentations, speakers bureaus, manuscript writing or educational events | ☒ **None** | |
| | | | |
| | | | |
| | | | |

| | | Name all entities with whom you have this relationship or indicate none (add rows as needed) | Specifications/Comments (e.g., if payments were made to you or to your institution) |
|---|---|---|---|
| **6** | Payment for expert testimony | ☒ **None** | |
| | | | |
| | | | |
| | | | |
| **7** | Support for attending meetings and/or travel | ☒ **None** | |
| | | | |
| | | | |
| | | | |
| **8** | Patents planned, issued or pending | ☒ **None** | |
| | | | |
| | | | |
| | | | |
| **9** | Participation on a Data Safety Monitoring Board or Advisory Board | ☒ **None** | |
| | | | |
| | | | |
| | | | |
| **10** | Leadership or fiduciary role in other board, society, committee or advocacy group, paid or unpaid | ☐ **None** | |
| | | Chair, Observational Study Monitoring Board, Diverse-VCID: White Matter Lesion Etiology of Dementia in Diverse Populations (Diverse VCID) Study, Bethesda, MD | 2021-present |
| | | Advisory Board Member, Lantheus – Diagnostic and Therapeutic Innovations, Billerica, MA | 2024-present |
| | | | |
| **11** | Stock or stock options | ☒ **None** | |
| | | | |
| | | | |
| | | | |
| **12** | Receipt of equipment, materials, drugs, medical writing, gifts or other services | ☒ **None** | |
| | | | |
| | | | |
| | | | |

| | | Name all entities with whom you have this relationship or indicate none (add rows as needed) | Specifications/Comments (e.g., if payments were made to you or to your institution) |
|---|---|---|---|
| **13** | Other financial or non-financial interests | ☒ **None** | |
| | | | |
| | | | |
| | | | |

**Please place an "X" next to the following statement to indicate your agreement:**

☒ I certify that I have answered every question and have not altered the wording of any of the questions on this form.
